# Preoperative MRI Assessment of Hamstring Tendons to Predict the Quadruple Hamstring Graft Diameter in Anterior Cruciate Ligament Reconstruction

**DOI:** 10.7759/cureus.21753

**Published:** 2022-01-31

**Authors:** Dhammapal S Bhamare, Saikishan Sirasala, Purvam Jivrajani, Abhishek Nair, Shubham Taori

**Affiliations:** 1 Department of Orthopaedics, Dr. D. Y. Patil Medical College, Hospital and Research Centre, Dr. D. Y. Patil Vidyapeeth, Pimpri, Pune, IND

**Keywords:** gracilis, semitendinosus, preoperative assessment, mri, quadruple hamstring graft diameter, acl reconstruction

## Abstract

Introduction

The cross-sectional area (CSA) and length of an individual's hamstring tendons are both variable, making it challenging for the operating surgeon to generate an ideal size graft during arthroscopic anterior cruciate ligament (ACL) reconstruction surgery. If we can predict the hamstring graft diameter using MRI (a routine radiological investigation used to diagnose knee pathology), this information, if obtained before surgery rather than after the harvesting of the hamstring tendons, may influence the graft choice and allow us to successfully perform ACL reconstruction with sufficient graft diameter.

Aims

The aims of this study were to determine the reliability and accuracy of 3T MRI in predicting quadruple hamstring graft diameter for ACL reconstruction, to determine the statistical correlation between the CSA of hamstring tendons on MRI and intraoperative quadruple hamstring graft diameter, and to find out the minimum CSA of hamstring tendons (ST+GR) required for an 8 mm quadruple hamstring graft diameter.

Methods

This prospective diagnostic study included 50 patients diagnosed with complete ACL rupture. On MRI, we assessed the CSA of the semitendinosus and gracilis tendons at two levels: one at the joint line and the other at the level where the anteroposterior (AP) diameter of the medial femoral condyle is longest. The quadruple ST+GR graft was passed through 0.5 mm increments of holes in the block (Biotek (Winooski, Vermont) or Smith & Nephew, London, United Kingdom) intraoperatively, and the diameter of the hole that permitted smooth passage of the whole graft was taken as the quadruple graft diameter. The cutoff CSA required for a graft of sufficient size was calculated using simple logistic regression analysis. The correlation between CSA measurements on MRI and intraoperative quadruple hamstring graft diameter was determined using Pearson's rank correlation coefficient.

Results

The mean ST+GR CSA on MRI was 18.9 mm^2^, the minimum CSA was 14.45 mm^2^, and the maximum CSA was 23.8 mm^2^. Pearson's correlation between the intraoperative quadruple hamstring graft diameter (mm) and ST+GR CSA on MRI was 0.838. The minimum ST+GR CSA required for an 8 mm quadruple hamstring graft diameter is 17.5 mm^2^.

Conclusion

A strong statistical correlation between ST+GR CSA on MRI and intraoperative quadruple hamstring graft diameter was found (Pearson's correlation = 0.838, p-value = 0.000). Thus, MRI is a reliable radiological investigation that can be used to predict the quadruple hamstring graft diameter. This method can help orthopedic surgeons successfully perform ACL reconstruction surgery without any graft complications.

## Introduction

The anterior cruciate ligament (ACL) is crucial for stabilizing the knee joint. The ACL prevents excessive forward tibial movement and internal tibial rotation [1-2]. ACL ruptures are a frequent sports injury in orthopedic practice due to their involvement in a multitude of activities [3-4]. Whether complete or partial, an ACL rupture is a serious ligament injury. This injury is quite prevalent in young people, especially sportsmen and those who live an active lifestyle, as it usually happens when playing sports like football, soccer, basketball, or skiing [5-6]. Due to the availability of improved health care, arthroscopic ACL reconstruction surgery has become increasingly popular in recent years, with over 10,000 ACL reconstructions performed each year in most developed and developing nations.

Graft reconstruction is now the most popular and recommended method for restoring ACL function and preserving knee stability. It can be an autograft or an allograft [7]. The graft choice depends on the individual's body build, the type of fixation devices to be utilized, and most importantly, the availability of the autograft or allograft to reconstruct the ACL.

In recent years, hamstring tendons, peroneus longus, patellar tendon, and quadriceps tendon have been the most widely utilized grafts globally [7]. In recent years, hamstring tendons have been the most popular and recommended autograft in ACL reconstruction. Compared to bone-patella-bone autografts, they have been shown to provide greater stability, stiffness, tensile characteristics, cosmesis, and functional results without donor site morbidity. The cross-sectional area and length of an individual's hamstring tendons are variable, making it difficult for the operating surgeon to prepare an ideal-sized graft during arthroscopic ACL reconstruction surgery. If the harvested tendon graft is inadequate in size, the operating surgeon may need to augment the graft or utilize other autografts to achieve a suitable graft size for ACL reconstruction (with the risk of graft failure if an undersized graft is used). Anthropometric measurements and radiographic investigations, such as MRI and ultrasound, are among the methods used to assess hamstring autograft sizes before surgery [8-10]. As a result, determining the graft to utilize in ACL reconstruction surgery will be aided by a preoperative assessment of the hamstring tendons. It is nearly possible to determine the graft diameter when using patellar or quadriceps tendon for ACL reconstruction surgery. However, if the chosen graft is hamstring tendons, it is not possible to accurately predict the graft size preoperatively [10]. If we can predict the hamstring graft diameter using MRI (effective radiological investigation), such information, if obtained before the surgery rather than after the harvesting of the hamstring tendons, may impact the choice of graft in order to successfully perform ACL reconstruction with sufficient graft diameter. ACL reconstruction with a less than 8 mm graft diameter has a high failure rate [11-13]. Thus, graft size is also significant in addition to the proper and adequate technique to improve the chances of success in ACL reconstruction surgery. Several efforts have been made to estimate the graft diameter using anthropometry, but this method has shown inconsistent results. Therefore, in recent studies, direct imaging approaches like MRI and USG have been used to estimate the graft diameter with more appropriate results.

In this prospective study, we aimed to perform a preoperative assessment of hamstring tendons (semitendinosus and gracilis) using magnetic resonance imaging. The measurements were validated and correlated with those documented during an ACL tear's surgical reconstruction. Based on the observation and results of this present prospective study, we were able to determine the ability of MRI to predict autografts of sufficient size in arthroscopic ACL reconstructions. We hypothesized that musculoskeletal radiologists and orthopedic surgeons, with combined efforts, would be able to estimate the graft size based on MRI findings.

## Materials and methods

This prospective diagnostic study was approved by the Institutional Ethics Sub-Committee (IESC) of Dr. D.Y. Patil Medical College and Research Centre in Pune (research approval number: IESC/PGS/2019/169). We conducted this study on 50 patients admitted to the department of orthopedics, DPU, Pune, diagnosed with primary complete ACL rupture, and informed written consent was taken from all 50 patients. This study was conducted between June 2019 and July 2021. Patients who were all fit for surgery were scheduled for arthroscopic reconstructive surgery of the ACL rupture after pre-anesthesia check-ups.

Inclusion criteria

Patients who have been diagnosed with a primary complete ACL tear and are more than 18 years of age but less than 60 years of age were included in this study.

Exclusion criteria 

Patients who were medically unfit for surgery, patients who had failed primary ACL reconstruction surgery, patients who had multi-ligament injuries to the knee joint in addition to the ACL tear, and patients who had semitendinosus and/or gracilis tendon injuries were all excluded from the study.

Preoperative MRI assessment of hamstring tendons

Prior to surgery, the semitendinosus and gracilis tendons were assessed using MRI. All the scans were conducted by a single musculoskeletal radiologist and measurements were recorded. The hamstring tendons were analyzed preoperatively on multiple views of MRI for all patients in the study, and the CSA was documented before surgery by orthopedic surgeons involved in the study. The radiologist who participated in this study was not informed of the predicted values for quadruple graft size. The following were the imaging specifications for the MRI system used in this study. The model used was MAGNETOM Siemens Vida (Siemens Healthineers, Erlangen, Germany), magnetic strength: 3 Tesla, planes: axial and sagittal; and the sequences were: Proton density/T2 weighted with fat saturation.

**Figure 1:**
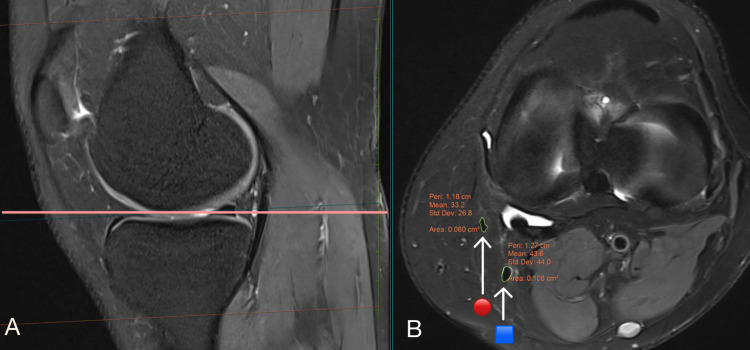
MRI scans showing (A) a sagittal view of the right knee joint; (B) an axial view of the right knee joint at the joint line, displaying the cross-sectional area of the semitendinosus and gracilis 🟦 denotes semitendinosus tendon; 🔴 denotes gracilis tendon

**Figure 2:**
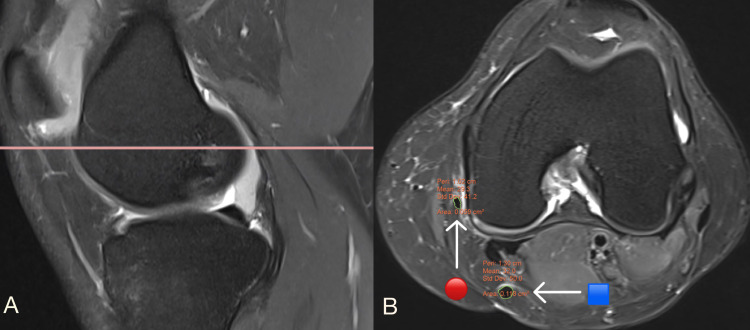
MRI scans showing (A) a sagittal view of the right knee joint; B) an axial view of the right knee joint at a level where the AP diameter of the medial femoral condyle is longest 🟦 denotes semitendinosus tendon; 🔴 denotes gracilis tendon

The freehand area of interest tool in the picture archiving and communication system (PACS) was used for MRI measurements. In this study, we used sagittal and axial views on MRI to measure the CSA of the hamstring tendons (ST+GR), one at the joint line (Figures 1A-1B) and the other at the level where the AP diameter of the medial femoral condyle is longest (Figures 2A-2B). At these two levels, the CSA of hamstring tendons was maximum, and the CSA was measured in axial views at the highest magnification where individual pixels could be seen [14]. The CSA of tendons is unaffected by MRI magnification, which is relevant to both 3 Tesla and 1.5 Tesla MRI [14]. The MRI measurements were correlated with the intraoperative quadruple hamstring graft diameter.

Operative procedure

**Figure 3:**
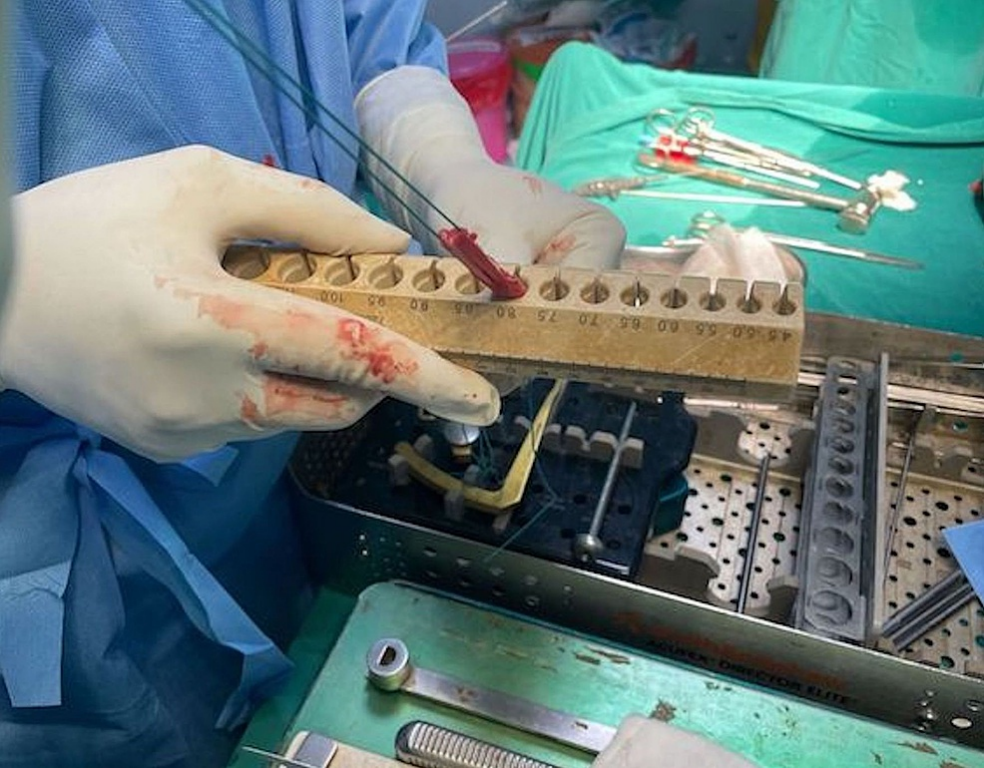
The intraoperative diameter of the quadruple hamstring graft was measured with the help of a sizing cylinder

An orthopedic surgeon harvested the semitendinosus and gracilis from the injured lower limb using a standard short linear incision of 3 cm length at the distal attachment of the tendons. The muscle fibers were removed and clipped after the tendons were harvested as part of the graft preparation. The lengths of the gracilis and semitendinosus were measured and documented intraoperatively using a sterilized standard metric stainless steel scale. To construct a quadruple hamstring graft, the ends of harvested tendons were stitched using polyester sutures (ethibond or fiber wire), and both tendon grafts were folded once in the center. In 0.5 mm increments, the graft was passed through the holes of the block (Biotek (Winooski, Vermont) or Smith & Nephew (London, United Kingdom)), and the diameter of the hole that facilitated seamless passage of the complete graft was taken as the quadruple graft diameter (Figure 3). Drill bits were used to drill the femoral and tibial tunnels appropriate to the diameter of the quadruple hamstring graft for anatomical single-bundle arthroscopic ACL reconstruction. The quadruple hamstring graft was secured with an endobutton at the femur end and the peak/bio/titanium screw at the tibial end in all patients, and the screw was chosen depending on patient affordability, nature of the ACL rupture, and patient preference. After the ACL reconstruction surgery with hamstring graft was completed, the operated knee joint was thoroughly lavaged with normal saline to remove any debris. Sutures were used to close all of the incision sites and portals. The dry dressing was placed, Gamjee rolls were used for compression, and a long knee extension brace was fitted. Postoperative pain management and rehabilitation were carried out as planned.

Statistical analysis

Statistical analysis of the data was performed by using SPSS version 20.0 (Released 2011. IBM SPSS Statistics for Windows, Version 20.0. Armonk, NY: IBM Corp). Quantitative data were presented as mean and standard deviation (SD). Pearson's rank correlation coefficient was used to determine the relationship between the two-scale variables. A two-tailed p-value of less than 0.005 was considered significant. A simple logistic regression analysis was used to determine the cutoff values for the ST+GR cross-sectional areas on MRI to prepare a quadruple hamstring graft with a diameter of 8 mm.

## Results

**Figure 4:**
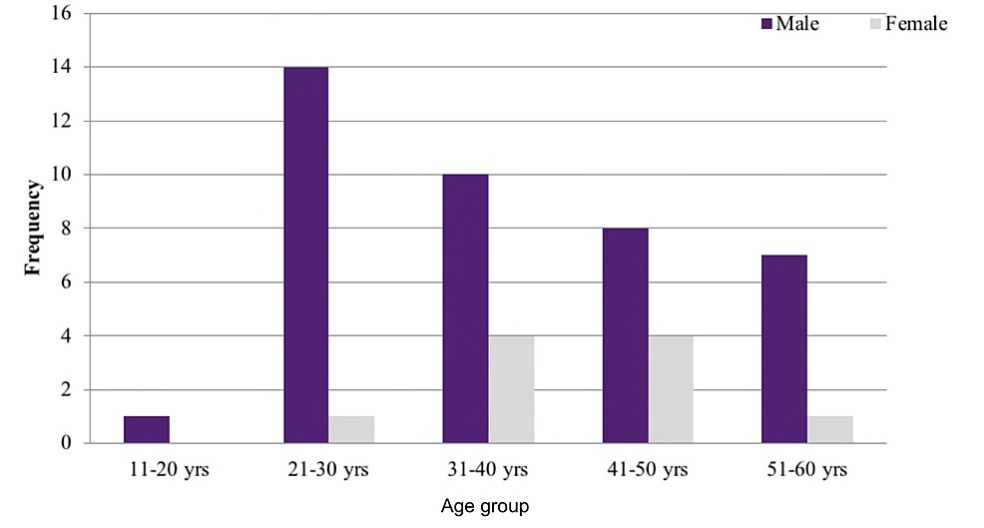
Bar chart showing age and gender distribution of patients included in this study (n = 50)

This prospective research study included 50 patients with a mean age of 37.86 years (SD = 10.99 years), with the highest being 58 years and the lowest being 18 years. There were 40 (80%) males and 10 (20%) females in the study (Figure 4).

**Figure 5:**
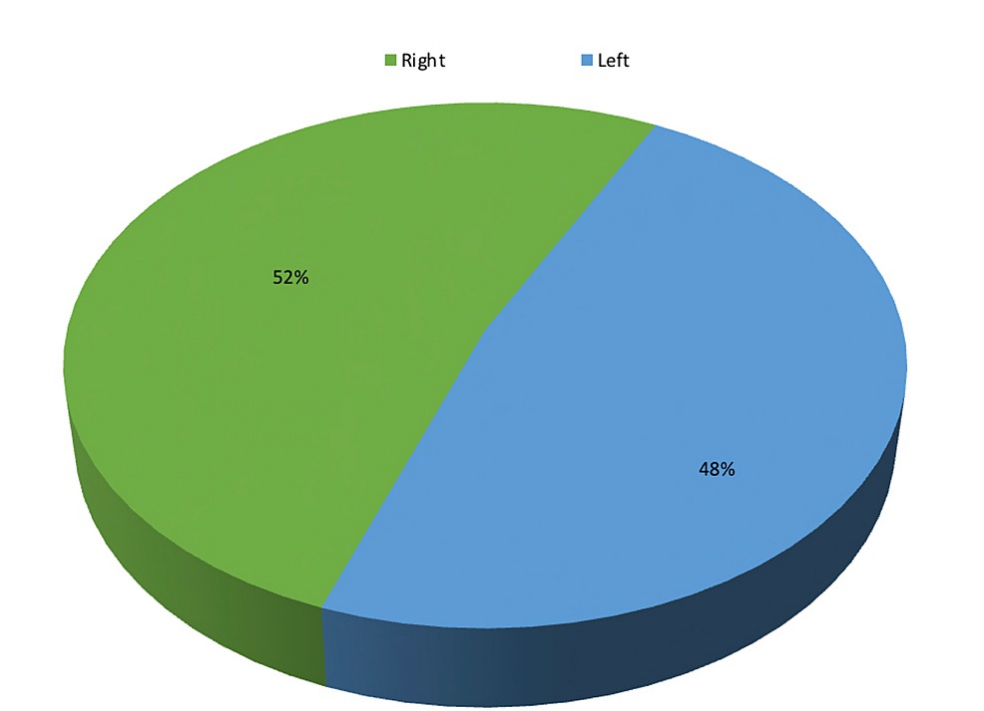
Pie chart showing the side of injury of patients included in this study (n = 50)

Twenty-six (52%) patients were injured on the right side while the remaining 24 (48%) were injured on the left side (Figure 5). The average period from injury to ACL reconstruction surgery in this study was 39.72 days, with a standard deviation of 16.74 days.

**Figure 6:**
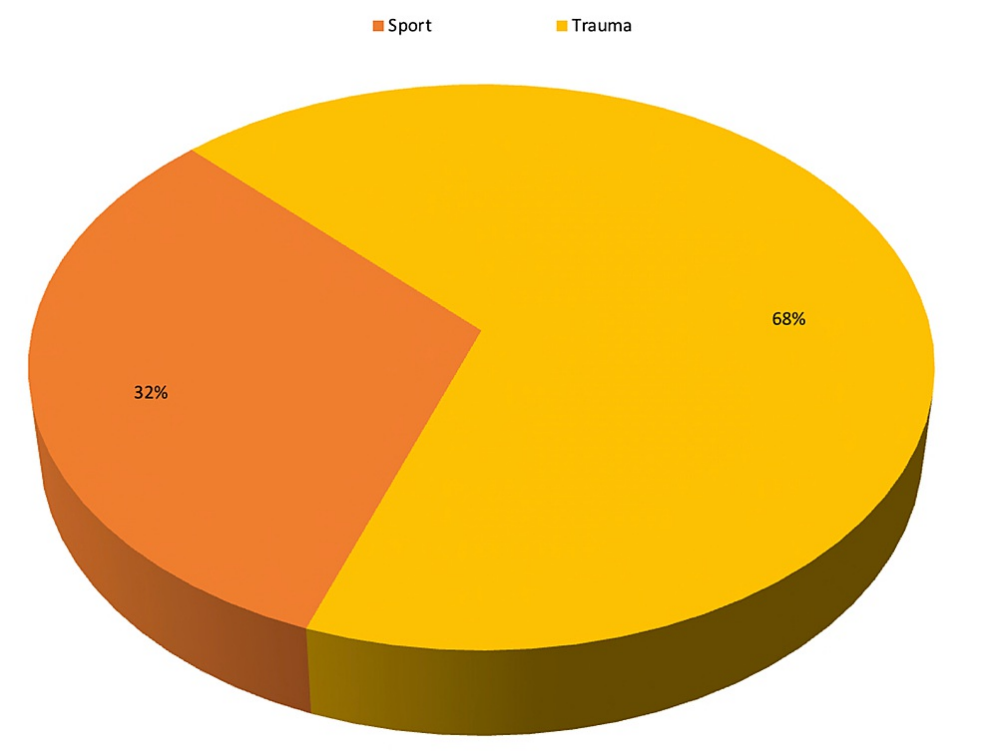
Pie chart showing the mode of injury of patients included in this study (n = 50)

Thirty-four (68%) patients were injured due to road traffic incidents while the remaining 16 (32%) were injured while participating in sports (Figure 6).

The mean semitendinosus + gracilis CSA (measured in the MRI axial view at the joint line and at a level where the AP diameter of the medial femoral condyle is longest) was 18.9 mm^2^, the minimum CSA was 14.45 mm^2^, and the maximum CSA was 23.8 mm^2^.

The mean intraoperative quadruple hamstring graft diameter was 8.64 mm, the minimum was 6.5 mm, and the maximum was 10.5 mm. The mean intraoperative semitendinosus graft length was 29.74 cm, the minimum was 23 cm, and the maximum was 40 cm. The mean intraoperative gracilis graft length was 27.36 cm, the minimum was 21 cm, and the maximum was 33 cm (Table 1).

**Table 1:** Intraoperative measurements of the hamstring tendon grafts

Intraoperative measurements	Mean	Range
Quadruple hamstring graft diameter	8.64 mm	6.5 mm - 10.5 mm
Semitendinosus graft length	29.74 mm	23 mm - 40 mm
Gracilis graft length	27.36 mm	21 mm - 33 mm

Among 50 patients, seven patients (14% of the study population) had a quadruple hamstring graft diameter of less than 8 mm, 30 patients (60% of the study population) had a quadruple hamstring graft diameter of between 8 mm and 9 mm, and 13 patients (26% of the study population) had a quadruple hamstring graft diameter of more than 9 mm (Table 2).

**Table 2:** The distribution of patients included in this study according to intraoperative quadruple hamstring graft diameter

Intraoperative quadruple hamstring graft diameter	Number of patients (n=50)
Less than 8 mm	7 (14%)
8 mm to 9 mm	30 (60%)
Greater than 9 mm	13 (26%)

**Figure 7:**
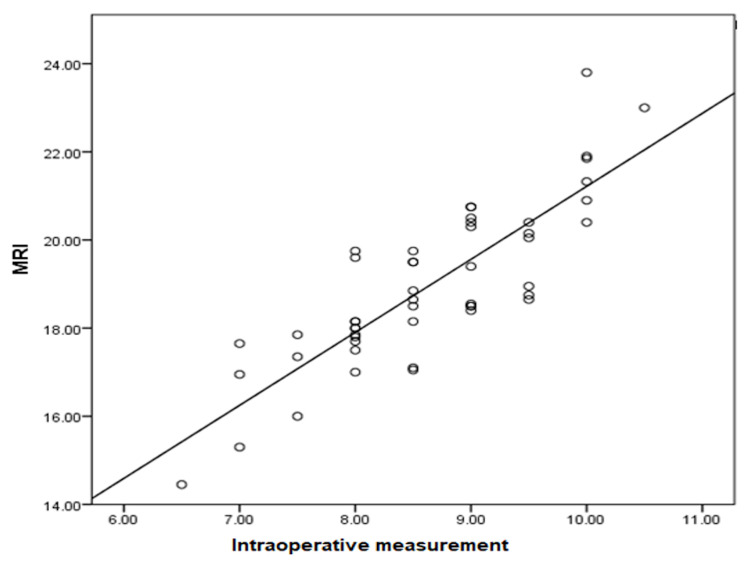
Scatter plot showing the correlation between the CSA of hamstring tendons (ST+GR) on MRI and the intraoperative quadruple hamstring graft diameter CSA: cross-sectional area; ST: semitendinosus; GR: gracilis; MRI: magnetic resonance imaging

In this prospective study, we found a very strong statistical relationship between the CSA of the semitendinosus and gracilis tendons on MRI and the true quadruple hamstring graft diameter noted during the ACL reconstruction surgery. Pearson's correlation between intraoperative quadruple hamstring graft diameter (mm) and ST+GR CSA on MRI was 0.838. According to this study's statistical results, MRI has a high statistical significance (Pearson's correlation = 0.838, p-value = 0.000) for predicting intraoperative quadruple hamstring graft diameter (Figure 7). As assessed on MRI, the minimal ST+GR cross-sectional area needed for an 8 mm quadruple hamstring graft diameter is 17.5 mm^2^. It is indeed 20.4 mm^2^ for a 10 mm quadruple hamstring graft.

## Discussion

Revision ACL surgeries after primary ACL reconstruction are reported to account for around 5% [15]. According to a few studies, 8%-9% of individuals who have surgery for an ACL injury will have graft failure, necessitating revision surgery [16]. The majority of graft failures are attributed to surgical mistakes, incorrect graft selection, and inadequate graft size. Magnussen et al. studied 265 patients who were all subsequently surgically treated with primary ACL reconstruction using hamstring tendon autograft, and it was observed that subjects in the younger age group and with graft diameters of less than 8 mm were risk factors for ACL revision surgery [17].

Many studies have been done to predict quadruple hamstring graft size using anthropometric equations and parameters, and many have analyzed the correlation between anthropometric parameters (height, weight, BMI, and thigh length) and quadruple hamstring graft diameter. Several authors have used radiological modalities, such as MRI, USG, and 3D CT, to determine the hamstring tendon graft size prior to the surgery. We found that imaging modalities like MRI and USG had a better correlation with intraoperative graft measurements than anthropometric parameters. The most important finding of this prospective study was that we found a very strong statistical relationship between the CSA of the ST and GR tendons on MRI and the intraoperative quadruple hamstring graft diameter. Pearson’s correlation between the intraoperative quadruple hamstring graft diameter (mm) and ST+GR CSA on MRI was 0.838.

Bickel et al. reported that the only level was used to measure CSA of ST and GR (level just distal to the physics) [8]. Their study was done in the adolescent age group so measuring at the physis was appropriate and suitable. The present study included adults with no specific level, such as the physis, for recording the CSA of hamstring tendons; this led to using two different levels instead of a single level, increasing the accuracy of measuring CSA hamstring tendons on MRI. In this study, we used sagittal and axial views on MRI to measure the CSA of the hamstring tendons (ST+GR), one at the joint line (Figures 1A-1B) and the other at the level where the AP diameter of the medial femoral condyle is longest (Figures 2A-2B). Bickel et al. reported a strong statistical correlation between the combined CSA of ST+GR and the intraoperative quadruple hamstring graft diameter [8]. They also found that the minimum ST+GR CSA on MRI required to achieve a graft size of 8 mm was 18 mm^2^, and its probability was 88%.

Hanna et al. reported a strong statistical correlation between the combined CSA of semitendinosus (ST) + gracilis (GR) on MRI and intraoperative quadruple hamstring graft diameter (p < 0.001), and the overall interclass correlation coefficient (ICC) was 0.977 [18]. In their study, the minimum ST+GR CSA on MRI required to achieve a graft size of 8 mm was 17.16 mm^2^. Hollangel et al. reported a strong statistical correlation between the combined CSA of semitendinosus (ST) + gracilis (GR) on MRI and intraoperative quadruple hamstring graft diameter (p < 0.001) and the overall interclass correlation coefficient (ICC) was 0.96 [14]. In their study, the minimum ST+GR CSA on MRI required to achieve a graft size of 8 mm was 17.5 mm^2^. Hollangel et al. used two levels on MRI to measure the CSA of hamstring tendons; one at the point where the medial femoral condyle is widest and the other at the joint line [14]. Erquicia et al. compared MRI and USG in the prediction of hamstring graft size and concluded that MRI showed a higher statistical correlation (0.86) with intraoperative quadruple hamstring graft diameter, whereas USG showed a moderate correlation (0.51) [10]. A study based on cadaveric dissection done by Pagnani et al. showed similar CSA measurements of hamstring tendons on MRI, but no statistical correlation was observed with intraoperative quadruple graft diameter [19]. In the study done by Yasuda et al., a 3D CT scan showed no significant correlation between hamstring graft length, CSA of tendons, and intraoperative graft diameter [20]. Furthermore, a CT scan is not a routine radiological investigation for soft tissue injuries like an ACL rupture.

Tuman et al. conducted a retrospective study, which included 106 study samples with a female to male ratio of 55:51 [21]. They studied the correlation between various anthropometric parameters (height, weight, age, and gender), and they found a good correlation between intraoperative quadruple ST+GR graft diameter and the height of the study subjects (p < 0.001), whereas weight, age, and gender showed poor correlation [21]. Treme et al. reported a moderate statistical correlation (R^2^ = 0.36-0.41) between anthropometric parameters and intraoperative hamstring graft diameter [22]. Pinheiro et al. discovered a strong statistical correlation between the study subjects’ height (cm) and thigh length (cm) and the intraoperative quadruple hamstring graft diameter [23]. The minimum ST+GR CSA required to prepare a quadruple hamstring graft of 8 mm in diameter was 17.5 mm^2^. According to the data, although there is no significant difference in cutoff values among various studies, variability does exist. For example, Erquicia et al. reported a cutoff value of 25.5 mm^2^ for an 8 mm graft [10] while Leiter et al. reported a cutoff value of 14.5 mm^2^ for a 7.5 mm graft diameter [24]. Hollangel et al. reported a cutoff value of 18.2 mm^2^ and 17.5 mm^2^ for 1.5 T MRI and 3 T MRI groups, respectively [14]. Hanna et al. reported a cutoff value of 17.168 mm^2^. Wernecke et al. recommended a CSA of 27 mm^2^ of ST+GR on MRI for reliably predicting the graft diameter for double-bundle ACL reconstruction (p = 0.06) [25]. This variability is due to the different techniques of measuring the CSA of hamstring tendons on MRI and the difference in sample size, gender, and race of the study population. Hence, further research is required to lay down a standard protocol to determine the cutoff values.

Limitations

Standardized graft sizing equipment was used to measure the diameter and length of the tendon, and these measurements may not be accurate to micrometers. In this prospective study, a single musculoskeletal radiologist measured all the cross-sectional areas on MRI using the freehand region tool. Thus, in this study, inter-observer variation was not considered, and the efficacy of the freehand region tool has not been standardized yet.

## Conclusions

This study showed that ST+GR CSA on MRI and the intraoperative quadruple hamstring graft diameter had a strong statistical relationship. We conclude that MRI is a reliable radiological investigation that can be used to predict the quadruple hamstring graft diameter. Further, preoperative assessment of autografts provides better preoperative planning and graft selection options for ACL reconstruction. This method can assist orthopedic surgeons in performing ACL reconstruction surgery successfully and without graft complications. In this study, intraoperative quadruple hamstring grafts were correlated with MRI measurements, and it would be interesting to see whether MRI prediction works for other multistranded grafts as well.
